# 166. A Phase 2b/3 Study to Evaluate the Efficacy and Safety of an Investigational Respiratory Syncytial Virus (RSV) Antibody, Clesrovimab, in Healthy Preterm and Full-Term Infants

**DOI:** 10.1093/ofid/ofae631.003

**Published:** 2025-01-29

**Authors:** Heather J Zar, Eric Simoes, Sabhir Madhi, Octavio Ramilo, Shelly Senders, Julie S Shepard, Kamolwish Laoprasopwattana, Jorge Piedrahita, Jose M Novoa Pizarro, Sergio L Vargas, Marc Dionne, Teresa Jackowska, Enmei Liu, Yasunori Ishihara, Kazushige Ikeda, Ying Zhang, Radha A Railkar, Jeannine Lutkiewicz, Andrew W Lee, Andrea Guerra, Anushua Sinha

**Affiliations:** Red Cross Children's Hospital and SA-MRC Unit on Child & Adolescent Health, University of Cape Town, South Africa, Cape Town, Western Cape, South Africa; University of Colorado School of Medicine and Colorado School of Public Health, Denver, CO; Wits Vaccines & Infectious Diseases Analytics (VIDA) Research Unit, Johannesburg, Gauteng, South Africa; St. Jude Children's Research Hospital, Memphis, TN; Senders Pediatrics, Cleveland, Ohio, United States, Euclid, Ohio; Ohio Pediatric Research Association, Dayton, Ohio; Prince of Songkla University, Songkla, Pattani, Thailand; Clinica de la Costa, Barranquilla, Atlantico, Colombia; Hospital Padre Alberto Hurtado, Santiago, Region Metropolitana, Chile; University Chile School of Medicine, Santiago, Region Metropolitana, Chile; CHU de Quebec-Universite Laval, Quebec City, Quebec, Canada; Center of Postgraduate Medical Education & Bielanski Hospital, Warsaw, Mazowieckie, Poland; Children's Hospital of Chongqing Medical University, Chongqing, Chongqing, China; Fukui Aiiku Hospital, Fukui, Gifu, Japan; Saitama City Hospital, Saitama, Saitama, Japan; Merck & Co., Inc., Kenilworth, New Jersey; Merck & Co., Inc., Kenilworth, New Jersey; Merck & Co., Inc., Kenilworth, New Jersey; Merck & Co., Inc., present affiliation Uniquity Bio, Malvern, PA, USA, Rahway, New Jersey; Merck Sharp & Dohme LLC - United Kingdom, London, England, United Kingdom; Merck and Co Inc., Rahway, NJ

## Abstract

**Background:**

Clesrovimab is an investigational, long-acting monoclonal antibody (mAb) targeting site IV of the fusion protein for the prevention of RSV lower respiratory tract infection in infants.

**Figure:**
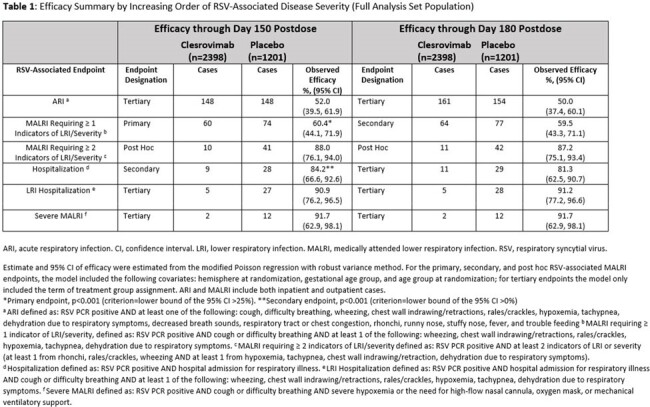

**Methods:**

This phase 2b/3 double-blind, randomized, placebo-controlled pivotal study enrolled healthy preterm and full-term infants birth to 1 year of age entering their first RSV season. Participants (pts) were randomized 2:1 to receive clesrovimab (105 mg IM) or placebo on day 1. Safety and tolerability were a primary endpoint. There were two hypothesis-tested endpoints: the efficacy of clesrovimab against RSV-associated medically attended lower respiratory tract infection (MALRI) through day 150 (primary) and against RSV-associated hospitalization through day 150 (secondary). The MALRI definition required ≥1 indicators of lower respiratory tract infection (LRI) or severity. To facilitate comparison across RSV mAb trials, a definition of RSV-associated MALRI that required ≥2 indicators of LRI/severity (≥1 indicator of LRI and ≥1 indicator of severity) was assessed post hoc.

**Figure:**
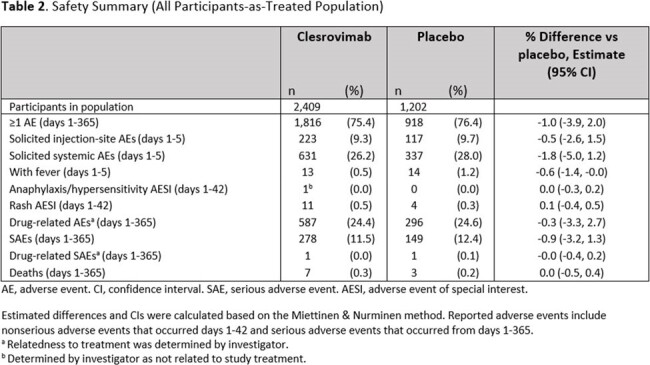

**Results:**

There were 3,632 pts randomized across 22 countries; >99% received study intervention. RSV-associated efficacy endpoints through day 150 and day 180 are shown in Table 1. Clesrovimab reduced the incidence of RSV-associated MALRI requiring ≥1 indicator of LRI/severity (60.4% [95% CI: 44.1, 71.9], p< 0.001) and ≥2 indicators of LRI/severity (88.0% [95% CI:76.1, 94.0]), RSV hospitalization (84.2% [95% CI: 66.6, 92.6], p< 0.001), and severe MALRI (91.7% [95% CI:62.9, 98.1]) through day 150 postdose compared to placebo. Efficacy increased with increasing RSV-associated disease severity and was similar from days 1-180 compared to days 1-150 across endpoints. The proportions of pts with adverse events (AEs), including injection-site and systemic AEs, drug-related AEs, and serious AEs were comparable between the clesrovimab and placebo groups (Table 2). There were no treatment-related deaths or deaths attributed to RSV disease.

**Conclusion:**

A single dose of clesrovimab given before or during the first RSV season was efficacious in reducing RSV-associated MALRI and RSV-associated hospitalization in healthy preterm and full-term infants and was generally well tolerated with a safety profile comparable to placebo.

**Disclosures:**

**Heather J. Zar, PhD**, MSD, Pfizer, AstraZeneca, Moderna (DSMB): Advisor/Consultant|MSD, Pfizer, AstraZeneca, Moderna (DSMB): Grant/Research Support|MSD, Pfizer, AstraZeneca, Moderna (DSMB): Honoraria|MSD, Pfizer, AstraZeneca, Moderna (DSMB): MSD Principal Investigator for the study and on MSD Advisory Board **Eric Simoes, MD DCH**, Pfizer, Sanofi, Merck, Icosavax, Enanta Cidara, Adiagio, Nuance, Shionogi, GIlead,: Advisor/Consultant|Pfizer, Sanofi, Merck, Icosavax, Enanta Cidara, Adiagio, Nuance, Shionogi, GIlead,: Grant/Research Support|Pfizer, Sanofi, Merck, Icosavax, Enanta Cidara, Adiagio, Nuance, Shionogi, GIlead,: Honoraria|Pfizer, Sanofi, Merck, Icosavax, Enanta Cidara, Adiagio, Nuance, Shionogi, GIlead,: MSD - PI for this study; DSMB Abbvie, Moderna, GSK **Sabhir Madhi, MD**, Pfizer, GSK, Medimmune, Merck & Co., Inc., Rahway, NJ, USA: Grant/Research Support|Pfizer, GSK, Medimmune, Merck & Co., Inc., Rahway, NJ, USA: Honoraria **Octavio Ramilo, MD**, Pfizer, Sanofi, Gates Foundation, NIH, and Merck Sharp & Dohme LLC, a subsidiary of Merck & Co., Inc., Rahway, NJ, USA (MSD): Advisor/Consultant|Pfizer, Sanofi, Gates Foundation, NIH, and Merck Sharp & Dohme LLC, a subsidiary of Merck & Co., Inc., Rahway, NJ, USA (MSD): Grant/Research Support|Pfizer, Sanofi, Gates Foundation, NIH, and Merck Sharp & Dohme LLC, a subsidiary of Merck & Co., Inc., Rahway, NJ, USA (MSD): Honoraria|Pfizer, Sanofi, Gates Foundation, NIH, and Merck Sharp & Dohme LLC, a subsidiary of Merck & Co., Inc., Rahway, NJ, USA (MSD): SAC member for MSD **Shelly Senders, MD**, Merck Sharp & Dohme LLC, a subsidiary of Merck & Co., Inc., Rahway, NJ, USA: Principal Investigator **Julie S. Shepard, MD, MPH**, Merck Sharp & Dohme LLC, a subsidiary of Merck & Co., Inc., Rahway, NJ, USA: Principal Investigator **Kamolwish Laoprasopwattana, MD**, Merck & Co., Inc., Rahway, NJ, USA: Grant/Research Support|Merck & Co., Inc., Rahway, NJ, USA: MSD - PI for this study **Jorge Piedrahita, MD**, Merck Sharp & Dohme LLC, a subsidiary of Merck & Co., Inc., Rahway, NJ, USA: Principal Investigator **Jose M. Novoa Pizarro, MD**, Merck Sharp & Dohme LLC, a subsidiary of Merck & Co., Inc., Rahway, NJ, USA (MSD): Principal Investigator for the study for MSD **Sergio L. Vargas, MD**, Merck Sharp & Dohme LLC, a subsidiary of Merck & Co., Inc., Rahway, NJ, USA: Principal Investigator **Marc Dionne, MD**, Merck Sharp & Dohme LLC, a subsidiary of Merck & Co., Inc., Rahway, NJ, USA: Principal Investigator **Teresa Jackowska, MD**, MSD Poland, Merck & Co., Inc, Rahway, NJ, USA: Advisor/Consultant|MSD Poland, Merck & Co., Inc, Rahway, NJ, USA: Grant/Research Support|MSD Poland, Merck & Co., Inc, Rahway, NJ, USA: Honoraria|MSD Poland, Merck & Co., Inc, Rahway, NJ, USA: MSD - PI for this study **Enmei Liu, MD**, Merck Sharp & Dohme LLC, a subsidiary of Merck & Co., Inc., Rahway, NJ, USA: Principal Investigator **Yasunori Ishihara, MD, PhD**, Merck Sharp & Dohme LLC, a subsidiary of Merck & Co., Inc., Rahway, NJ, USA: Principal Investigator **Kazushige Ikeda, MD**, Merck Sharp & Dohme LLC, a subsidiary of Merck & Co., Inc., Rahway, NJ, USA: Principal Investigator **Ying Zhang, PhD**, Merck Sharp & Dohme LLC, a subsidiary of Merck & Co., Inc., Rahway, NJ, USA: Employee|Merck Sharp & Dohme LLC, a subsidiary of Merck & Co., Inc., Rahway, NJ, USA: Stocks/Bonds (Public Company) **Radha A. Railkar, PhD**, Merck Sharp & Dohme LLC, a subsidiary of Merck & Co., Inc., Rahway, NJ, US (MSD): Employee|Merck Sharp & Dohme LLC, a subsidiary of Merck & Co., Inc., Rahway, NJ, US (MSD): Stocks/Bonds (Public Company) **Jeannine Lutkiewicz, BS**, Merck Sharp & Dohme LLC, a subsidiary of Merck & Co., Inc., Rahway, NJ, USA: Employee|Merck Sharp & Dohme LLC, a subsidiary of Merck & Co., Inc., Rahway, NJ, USA: Stocks/Bonds (Public Company) **Andrew W. Lee, MD**, Merck Sharp & Dohme LLC, a subsidiary of Merck & Co., Inc., Rahway, NJ, USA (MSD): Employee at the time of study|Merck Sharp & Dohme LLC, a subsidiary of Merck & Co., Inc., Rahway, NJ, USA (MSD): Stocks/Bonds (Public Company) **Andrea Guerra, MD**, Merck Sharp & Dohme LLC, a subsidiary of Merck & Co., Inc., Rahway, NJ, USA: Employee|Merck Sharp & Dohme LLC, a subsidiary of Merck & Co., Inc., Rahway, NJ, USA: Stocks/Bonds (Public Company) **Anushua Sinha, MD, MPH**, Merck Sharp & Dohme LLC, a subsidiary of Merck & Co., Inc., Rahway, NJ, USA (MSD): Employee|Merck Sharp & Dohme LLC, a subsidiary of Merck & Co., Inc., Rahway, NJ, USA (MSD): Stocks/Bonds (Public Company)

